# Risk factors and comorbidities associated with cardiac arrests and medical emergencies in interventional radiology patients

**DOI:** 10.1186/s42155-024-00504-z

**Published:** 2024-12-18

**Authors:** Husam Mohammed AlHarbi, Tarek Arabi, Yasser Saleh A. Alduribi, Hassan Shah, Ahmad Sabbah, Khalid Othman, Omar Bashir, Mohammad Arabi

**Affiliations:** 1 Division of Vascular and Interventional Radiology, Department of Medical Imaging, King Abdulaziz Medical City-Ministry of National Guard Health Affairs, Riyadh, Saudi Arabia; 2 College of Medicine, King Saud Bin Abdulaziz University for Health Sciences, Riyadh, Saudi Arabia; 3 King Abdullah International Medical Research Center, Riyadh, Saudi Arabia; 4 College of Medicine, Alfaisal University, Riyadh, Saudi Arabia; 5 Security Forces Hospital Program, Riyadh, Saudi Arabia

## Abstract

**Purpose:**

To investigate the incidence, predictors, and outcomes of medical emergencies in patients undergoing IR procedures at a tertiary care center.

**Materials and methods:**

Seven-year retrospective review of all medical emergencies in patients undergoing IR procedures at King Abdulaziz Medical City, Riyadh, Saudi Arabia. Medical emergencies included Cardiopulmonary arrest (CPA), or emergencies that required activation of the critical care response team (CCRT). Variables included demographics, procedure details and outcome data including complications and 30-day mortality. Multivariate logistic regression analysis was conducted to identify independent predictors of CPA and 30-day mortality.

**Results:**

Ninety-four patients (50% male) were included with a median age of 60.5 years. Recent or current ICU admission was recorded in 39 patients (43.8%). Comorbidities included diabetes (50%), hypertension (59.6%), coronary artery disease (25.5%), heart failure (21.5%), ESRD (28.7%), active infection 28 (31%), with ASA3 in 64 patients (68%) and ASA4 in 23 (24.5%).

The incidence of CPA and CCRT activation was 0.045% and 0.049%, respectively, among 100,000 patients who underwent IR procedures during the study period. Half the events were with venous procedures, followed by non-vascular (33%) and arterial procedures (10.6%). 30-day mortality was 30.5%. Independent predictors of CPA included pulmonary disease (aOR 16.79, 95% CI 2.334–195.3, *p* = 0.0097), emergency procedures (aOR 11.63, 95% CI 2.517–72.46, *p* = 0.0035), general anesthesia (aOR 19.41, 95% CI 1.854–491.8, *p* = 0.0254), and sedation (aOR 13.04, 95% CI 2.081–118.8, *p* = 0.0108). Predictors of 30-day mortality were CPA (aOR 9.830, 95% CI 2.439–66.66, *p* = 0.0045) and hypotension as a complication (aOR 16.81, 95% CI 3.766–122.3, *p* = 0.0009).

**Conclusion:**

Our findings highlight the complexity of patients undergoing IR procedures and the importance of identifying high-risk patients to prevent adverse events in the IR setting.

## Introduction

Interventional radiology (IR) has become an essential component of tertiary healthcare, offering a wide array of diagnostic and therapeutic procedures for various organ systems. IR allows for minimally invasive management of patients, including those who are critically ill in intensive care unit (ICU) or emergency room (ER) settings. Despite the less invasive nature of IR procedures compared to surgical interventions, adverse events such as cardiopulmonary arrest (CPA) and other medical emergencies can still occur.

A study from the National University Changwon Hospital in Korea analyzed 344,500 patients undergoing IR procedures and reported a CPA incidence rate of 0.0067%, which is ten times lower than that observed in surgical patients [1]. Another study from the University of Pennsylvania Medical Center, which included both CPA and medical emergencies, reviewed approximately 40,000 IR patients and reported an incidence of 0.15% for these events [2].

Several risk factors have been identified for the occurrence of medical emergency events during IR procedures. Chronic kidney disease, vascular procedures, and higher American Society of Anesthesiologists (ASA) classification have all been linked to a higher risk of CPA events [1, 2]. Additionally, patients with diabetes mellitus, hypertension, malignancies, and acute kidney injury are at least twice as likely to suffer CPA [1].

Mortality following CPA or medical emergencies in IR is a significant concern. Factors associated with increased mortality in IR patients include arterial procedures and pre-existing coronary artery disease (CAD) [1].

Identifying patients at higher risk for complications and understanding the outcomes of different IR procedures is crucial for patient safety and quality improvement. There are limited data on medical emergencies in IR settings specific to Middle Eastern or Saudi populations. This study addresses the gap in the literature by examining factors contributing to CPA and mortality in a Saudi tertiary care center, which may provide insights into regional variances in healthcare settings. We aim to investigate the association between IR procedures, CPA, and mortality, along with their associated risk factors in King Abdulaziz Medical City, Riyadh, Saudi Arabia.

## Materials and methods

### Study design and setting

This single-center retrospective study was conducted at the interventional radiology department of King Abdulaziz Medical City, Riyadh, Saudi Arabia. The study was approved by the institutional review board at King Abdulaziz Medical City, Riyadh, Saudi Arabia (protocol number NRC23R/226/04). Given the retrospective nature of the study, the requirement for informed consent was waived.

All patients who were age 14 years or older, underwent an interventional radiology procedure at King Abdulaziz Medical City, Riyadh, Saudi Arabia between Jan 2016 and June 2023, and experienced a CPA or required (CCRT) were included. Under hospital policy, patients aged 14 years or older are treated as adults; hence, all patients in that demographic were included in our analysis.

Study data were collected and managed using RedCap electronic data capture tools hosted at the Medical Imaging Department of King Abdulaziz Medical City, Riyadh, Saudi Arabia [3, 4]. Data were retrospectively retrieved from the electronic medical records and radiology information systems.

Variables included patient demographics (age, gender, comorbidities, baseline serology), procedure details (type of procedure, vascular or non-vascular, elective or emergency), and outcome data (occurrence of CPA or medical emergency, complications, 30-day mortality). Comorbidities were categorized based on established diagnostic criteria used in the electronic medical records system. Anesthesia types included moderate sedation administered by IR and general anesthesia, depending on the complexity of the procedure. CPA was defined as the sudden cessation of cardiac or respiratory function requiring immediate lifesaving measures. CCRT code was activated when specific institutional criteria are met.

Data were managed using Microsoft Excel and analyzed using SPSS software (version 25.0; IBM Corp., Armonk, NY, USA). Missing data were handled using the listwise deletion method, ensuring robustness in the final analysis. Categorical variables were described as frequencies and percentages. Continuous variables were reported as means with standard deviations or medians with interquartile ranges, as appropriate.

Univariate analysis was performed using the chi-square test or Fisher's exact test for categorical variables, and the Student's t-test or Mann–Whitney U test for continuous variables. Multivariate logistic regression analysis was conducted to identify independent predictors of CPA and 30-day mortality. Backward stepwise regression was used to select variables to be included in the multivariate model. Results were reported as adjusted odds ratios (aOR) with 95% confidence intervals (CI). A two-tailed *p*-value < 0.05 was considered statistically significant.

## Results

A total of 94 patients were included in the study, with an equal distribution of males and females (50% each). The median age of the patients was 60.5 years (IQR 30–90). The most prevalent comorbidities were hypertension, diabetes mellitus, and dyslipidemia, which were present in approximately half of the population (Table 1). ASA classification was ASA class 3 in 64 patients (68%) and ASA4 in 23 (24.5%). History of recent or current ICU admission was recorded in 39 patients (43.8%). Other comorbidities and demographics are summarized in Table 1.

**Table 1:** Characterstics of patients undergoing IR procedures who had a medical emergency

	**Population (Mean ± SD or *N* (%))**	**CPA (Mean ± SD or *N* (%))**	**CCRT (Mean ± SD or *N* (%))**	*P*-value*
**Number of patients**	94	45 (47.87%)	49 (52.13%)	
**Age (years)**	56.38 ± 20.36	53.40 ± 23.94	59.12 ± 16.17	0.3516 ​​
**Male**	47 (50%)	27 (60%)	20 (40.82%)	0.0631 ​​
**ASA classification**				
**ASA1**	1 (1%)	1 (1%)	0	0.4022
**ASA2**	5 (5.3%)	1 (1%)	4 (4.3%)	
**ASA3**	64(68%)	32(34%)	32 (34%)	
**ASA4**	23 (24.5%)	10 (10.6%)	13 (13.8%)	
**ASA5**	1 (1%)	1 (1%)	0	
**Diabetes mellitus**	46 (50%)	23 (52.27%)	23 (47.92%)	0.6764
**Hypertension**	56 (59.57%)	31 (68.89%)	25 (51.02%)	0.0778
**Dyslipidemia**	46 (49.46%)	24 (54.55%)	22 (44.90%)	0.3528
**Coronary artery disease**	24 (25.53%)	15 (33.33%)	9 (18.37%)	0.0964
**Heart failure**	20 (21.51%)	10 (22.73%)	10 (20.41%)	0.7858
**ESRD**	27 (28.72%)	16 (35.56%)	11 (22.45%)	0.1606
**Pulmonary disease**	17 (18.48%)	13 (29.55%)	4 (8.33%)	**0.0088**
**Liver cirrhosis**	5 (5.38%)	0 (0%)	5 (10.20%)	0.0294
**Malignancy**	9 (9.78%)	2 (4.55%)	7 (14.58%)	0.1055
**History of ICU admission**	39 (43.82%)	27 (65.85%)	12 (25%)	**0.0001**
**Infection**	28 (31.11%)	20 (47.62%)	8 (16.67%)	**0.0016**
**Elective procedure**	68 (72.34%)	28 (62.22%)	40 (81.63%)	**0.0356**
**Emergency procedure**	26 (27.66%)	17 (37.78%)	9 (18.37%)	**0.0356**
**ICU/ER as referring service**	23 (24.73%)	19 (43.18%)	4 (8.16%)	** < 0.0001** ​​
**Moderate sedation by IR**	11 (11.70%)	8 (17.78%)	3 (6.12%)	**0.0006 ​​**
**General anesthesia**	10 (10.64%)	9 (20.00%)	1 (2.04%)	**0.0006 ​​**
**Activation time**				
Pre-procedural	17 (18%)	6 (6.4%)	11 (11.7%)	0.2662
Intra-procedural	42 (44.7%)	20 (21.3%)	22 (23.4%)	
Post-procedural	19 (20.2%)	16(17%)	35 (37.2%)	
**Contrast used**	56 (61.54%)	27 (62.79%)	29 (60.42%)	0.8162 ​​
**Venous procedures**	47 (50%)	28 (62.22%)	19 (38.78%)	**0.0112 ​​**
**Non-vascular procedures**	31 (32.98%)	8 (17.78%)	23 (46.94%)	**0.0389 ​​**
**Arterial procedures**	10 (10.64%)	7 (15.56%)	3 (6.12%)	**0.0112** ​​

^*^bolded *p*-values are statistically significant

The majority of procedures were elective (72.3%), with 75.3% of patients referred from the general ward. The rate of CPA in elective procedures was 41.2%, while the rate in emergency procedures was 65.4%. Local anesthesia was the most common (74.5%), and contrast was used in 61.5% of procedures. Code was activated prior to the commencement of procedure in 17 (18%), while it was activated during the procedure in 42 (44.7%) and post-procedural in 19 (20.2%) (Table 1) Venous procedures included placement of various types of venous catheters, dialysis interventions, venous and pulmonary catheter directed therapeutic interventions. Arterial interventions included peripheral vascular interventions and arterial embolization procedures. Non-vascular procedures included biopsies and drain placements, and other similar non-vascular IR procedures.

CPA events occurred in 45 (47.87%) patients, while CCRT activation was required in 49 (52.13%). The most common procedures were venous (50%), followed by non-vascular (32.98%) and arterial (10.64%). During the study period, approximately 100,000 patients underwent IR procedures, resulting in an incidence rate of 0.045% for CPA events and 0.049% for CCRT activation. 20 CPA (44%) and 9 CCRT (18%) patients suffered from 30-day mortality (*p* = 0.0072), leading to a 30.53% mortality rate in our study sample.

### Factors associated with CPA

Cardiac arrhythmia (35.56% vs. 6.25%, *p* = 0.0006), pulmonary disease (29.55% vs. 8.33%, *p* = 0.0141), history of cardiac arrest (13.95% vs. 0%, *p* = 0.0085), history of ICU admission (65.85% vs. 25%, *p* = 0.0001), infection (47.62% vs. 16.67%, *p* = 0.0027), emergency procedures (37.78% vs. 18.37%, *p* = 0.04), and referral from ICU/ER (43.18% vs. 8.16%, *p* < 0.0001) were significantly associated with CPA events (Table 2). Patients undergoing procedures with sedation by anesthesia (6.67% vs. 0%) or general anesthesia (20% vs. 2.04%, *p* = 0.0002) had a higher incidence of CPA compared to those with moderate sedation. Non-vascular procedures (17.78% vs. 46.94%, *p* = 0.008) were associated with a lower risk of CPA events.

**Table 2:** The difference in laboratory parametrs between CCRT and CPA patients in our population

	**CCRT**	**CPA**	*P*-value*
Hemoglobin (g/L)	103 (90–121)	89 (77–106)	**0.0057**
INR	1.1 (1.04–1.26)	1.13 (1.06–1.3)	0.6219
Partial thromboplastin time (Seconds)	28.3 (26.2–32.8)	29.9 (26.5–38.4)	0.1061
Platelet count (X 10^9^/L)	195 (157–295)	304 (172–425)	**0.0457**
WBC (X 10^9^/L)	6.9 (4.9–9.28)	9.95 (8.94–19.3)	** < 0.001**
C-reactive protein (mg/L)	70 (30.02–93)	108 (12–132)	0.3349
Procalcitonin (ng/L)	0.16 (0.04–0.7)	0.5 (0.35–1.87)	0.204
Erythrocyte sedimentation rate (mm/hour)	42 (17–73)	46 (34–60)	0.9501
Sodium (mmol/L)	136 (133–139)	135 (131–139)	0.3977
Potassium (mmol/L)	4.3 (3.9–4.6)	4.4 (3.7–4.7)	0.6327
Magnesium (mmol/L)	0.77 (0.71–0.88)	0.84 (0.76–0.95)	**0.0283**
Creatinine (umol/L)	115 (60–372)	93 (57–397)	0.7678
Brain natriuretic peptide (ng/L)	355.8 (130.3–398.4)	67 (23.7–271)	0.1233
eGFR (mL/min/1.73m^2^)	57 (12–111)	39 (12–115)	0.9419
Troponin-I (ng/L)	31.6 (10.6–45.4)	16.9 (9.99–53)	0.4444
Lactate (mmol/L)	1.4 (0.89–2.41)	1.95 (1.34–2.51)	0.1323

^*^bolded *p*-values are statistically significant

**Fig. 1:**
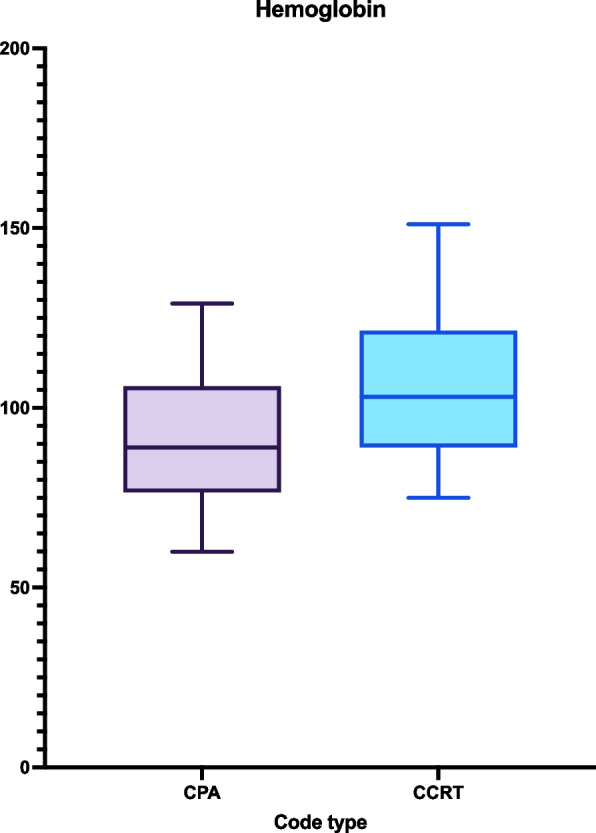
CPA patients have significantly lower hemoglobin levels compared to CCRT (*p* = 0.0057)

**Fig. 2:**
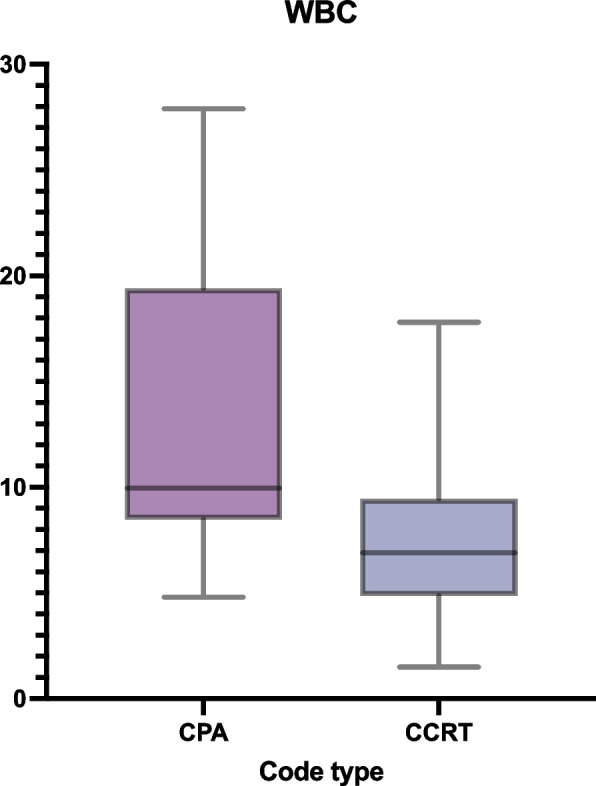
CPA patients have significantly higher WBC levels compared to CCRT (*p* = 0.0283)

In terms of laboratory parameters, CPA patients had lower hemoglobin levels (89 vs. 103 g/L, *p* = 0.005) (Fig. 1), higher platelet count (304 vs. 195 × 10^9^/L, *p* = 0.04), higher white blood cell (WBC) count (9.95 vs. 6.9 × 10^9^/L, *p* < 0.001) (Fig. 2). Other values, including PTT, procalcitonin, eGFR, and other electrolytes, were not different between the two groups (Table 2).

In the multivariate analysis, pulmonary disease (aOR 16.79, 95% CI 2.334–195.3, *p* = 0.009), emergency procedures (aOR 11.63, 95% CI 2.517–72.46, *p* = 0.003), general anesthesia (aOR 19.41, 95% CI 1.854–491.8, *p* = 0.0254), sedation (aOR 13.04, 95% CI 2.081–118.8, *p* = 0.01), and higher white blood cell count (aOR 1.268, 95% CI 1.087–1.532, *p* = 0.006) were independent predictors of CPA events. Non-vascular procedures (aOR 0.1365, 95% CI 0.01810–0.7183, *p* = 0.02) were associated with a lower risk of CPA compared to venous procedures (Table 3).

**Table 3:** Predictors of CPA in IR patients

Risk factors	aOR	95% CI	*P*-value
**History of cardiac arrhythmia**	6.326	0.8547 to 67.16	0.0876
**Pulmonary disease**	16.79	2.334 to 195.3	**0.0097**
**Emergency procedure**	11.63	2.517 to 72.46	**0.0035**
**Anesthetics**			
Local anesthesia	reference		
General anesthesia	19.41	1.854 to 491.8	**0.0254**
Sedation	13.04	2.081 to 118.8	**0.0108**
**Type of procedure**			
Venous	reference		
Arterial	0.7651	0.07139 to 7.776	0.8187
Non-vascular	0.1365	0.01810 to 0.7183	**0.0298**
**WBC**	1.268	1.087 to 1.532	**0.0060**
**Magnesium**	1.098	1.007 to 21.05	0.5590

^*^bolded *p*-values are statistically significant

### Predictors of 30-day mortality

Univariate analysis showed that coronary artery disease (41.38% vs. 18.46%, *p* = 0.02), heart failure (34.38% vs. 15.63%, *p* = 0.05), pulmonary disease (31.03% vs. 12.7%, *p* = 0.04), infection (48.28% vs. 22.95%, *p* = 0.02), emergency procedures (44.83% vs. 20%, *p* = 0.02), referral from ICU/ER (39.29% vs. 18.46%, *p* = 0.03), and CPA events (68.97% vs. 38.46%, *p* = 0.007) were significantly associated with 30-day mortality. Lower platelet count (median 240.50 vs. 183.00, *p* = 0.03), higher white blood cell count (median 8.64 vs. 11.60, *p* = 0.01), and higher troponin I levels (median 16.10 vs. 45.21, *p* = 0.0316) were also associated with increased mortality. In the multivariate analysis, CPA events (aOR 9.830, 95% CI 2.439–66.66, *p* = 0.004) and hypotension as a complication (aOR 16.81, 95% CI 3.766–122.3, *p* = 0.0009) were independent predictors of 30-day mortality (Table 4).

**Table 4:** Predictors of 30-day mortatlity in IR patients

Risk factor	aOR	95% CI	*P*-value
Pulmonary disease	3.422	0.9383 to 14.38	0.0724
Contrast used	3.282	0.9986 to 12.65	0.0625
CPA	9.830	2.439 to 66.66	**0.0045**
Hypotension as a complication	16.81	3.766 to 122.3	**0.0009**

^*^bolded *p*-values are statistically significant

## Discussion

This study investigates the incidence and predictors of CPA and medical emergencies during IR procedures at King Abdulaziz Medical City, Riyadh, Saudi Arabia. Our findings indicate CPA incidence rate of 0.045% across 7 years, which is within the range reported in the previous literature. For example, Nam et al. reported a CPA incidence rate of 0.0067% in a large-scale study at the National University Changwon Hospital in Korea [1]. This significant difference may be attributed to variations in patient demographics, procedural complexities, and healthcare systems. Conversely, Rueb et al. found a higher incidence rate of 0.15% in a study involving approximately 40,000 IR patients at the University of Pennsylvania Medical Center, underscoring procedural and institutional variability [2]. Nadolski et al. similarly reported a CPA rate of 0.15% during 38,927 procedures, with a 33% mortality rate at 30 days, comparable to our findings [5].

Several significant risk factors for CPA were identified in our study, including pulmonary disease, emergency procedures, general anesthesia, sedation, and elevated white blood cell count. These findings are consistent with those of Nam et al., who identified chronic kidney disease and higher ASA classification as significant predictors of CPA [1]. Similarly, Hope et al. reported that body mass index and the type of radiologic procedure significantly impacted survival to discharge following a CPA event [6].

Our analysis revealed that non-vascular procedures were associated with a lower risk of CPA, suggesting the nature of the procedure significantly influences the likelihood of adverse events. This compares with the findings of Rueb et al. [2], where dialysis shunt interventions had a higher relative risk of CPA compared to arterial interventions. The study by Nadolski et al. indicated that the relative risk for ME or CPA occurring during a hemodialysis access procedure versus other procedures was 5.2 (95% CI = 3.02–8.95; *P* < 0.0001). [5] Other studies have shown that vascular procedures carry higher risks due to their complexity and the underlying comorbidities of the patients involved [7, 8]. It is also likely that patients with lower severity of illness required less complex vascular procedures, which may have contributed to the lower CPA rates observed in our cohort.

The mortality rate following CPA or medical emergencies in IR remains a significant concern. Our study found that CPA, hypotension, and pre-existing pulmonary disease were independent predictors of 30-day mortality. These results align with broader literature, where factors such as coronary artery disease, heart failure, and infections have been linked to higher post-CPA mortality rates [9, 10]. Furthermore, a study by Siskovic et al. reported that type 2 diabetes mellitus significantly increases the risk of sudden cardiac arrest in the community [11].

Additionally, the survival rate of CPA patients in our study was relatively similar compared to other studies. Nam et al. reported a 73.9% survival rate until hospital discharge for CPA patients [1], which is higher than the 60.7% survival rate reported by Rueb et al. [2]. This could be attributed to differences in patient management and healthcare infrastructure.

Several studies have highlighted the importance of early identification and management of high-risk patients to improve survival outcomes. For example, Larkin et al. found that pre-resuscitation factors, such as patient comorbidities and initial rhythm, significantly impact survival rates following in-hospital cardiac arrest [12]. Similarly, Peberdy et al. noted that survival rates from in-hospital cardiac arrest were significantly lower during nights and weekends, suggesting the need for continuous and efficient monitoring systems [13].

From a clinical perspective, the identification of high-risk patients is vital for optimizing outcomes, particularly in resource-limited settings. Enhanced pre-procedural assessments and closer monitoring during and after IR procedures could mitigate adverse outcomes. For example, implementing early warning systems and standardizing sedation protocols may significantly improve patient safety. However, the retrospective and single-center nature of our study limits its generalizability. Potential biases from missing data and the exclusion of certain patient demographics further restrict the broader applicability of our findings. Future multicenter prospective studies are needed to validate these observations and improve the understanding of CPA risk factors in IR.

In conclusion, our study provides crucial insights into the incidence and predictors of CPA and medical emergencies during IR procedures in a tertiary care setting. By identifying key risk factors, we can enhance pre-procedural assessments and optimize perioperative care, ultimately improving patient safety and outcomes.

## Data Availability

Data will be made available on reasonable request.
